# From womb to world: mapping gut microbiota-related health literacy among Italian mothers, a cross-sectional study

**DOI:** 10.1186/s12889-024-18497-8

**Published:** 2024-04-12

**Authors:** Alessandra Consales, Letizia Toscano, Chiara Ceriotti, Valentina Tiraferri, Silvana Castaldi, Maria Lorella Giannì

**Affiliations:** 1 Department of Clinical Sciences and Community Health, University of Milan, Milan, Italy; 2 Department of Biomedical Sciences for Health, University of Milan, Milan, Italy; 3 ASST Grande Ospedale Metropolitano Niguarda, Milan, Italy; 4 Quality Unit, Fondazione IRCCS Ca’ Granda Ospedale Maggiore Policlinico, Milan, Italy; 5 NICU, Fondazione IRCCS Ca’ Granda Ospedale Maggiore Policlinico, Milan, Italy

**Keywords:** Gut microbiota, Dysbiosis, Health literacy, eHealth literacy, The first 1000 days, Pregnant women, Mothers, Breastfeeding

## Abstract

**Background:**

The gut microbiota is a key determinant of long-term health. Promoting maternal health literacy may enhance children well-being. Aim of the present study was to assess gut microbiota-related health literacy of Italian women and identify potential gaps in awareness.

**Methods:**

A cross-sectional survey study was conducted using an online questionnaire (17 questions) on determinants and long-term impact of infant gut microbiota. The survey targeted Italian pregnant women and mothers of children under 2 years old, and was distributed through various social media channels between September 28th and November 15th, 2022. A total score was calculated as the sum of positive answers. Data on demographics, pregnancy status, and pre-existing knowledge of the infant gut microbiota were also collected. Descriptive and inferential statistics were applied.

**Results:**

The questionnaire was completed by 1076 women. Median total score was 9 [7–11]. The 81.7% of respondents declared prior knowledge of the gut microbiota. The internet was among the most commonly cited primary sources of information. Independent predictors of total score were having a university degree (B = 0.656, *p* = 0.002) and prior knowledge (B = 2.246, *p* < 0.001). Conversely, older age was associated with lower total scores (B = -0.092, *p* < 0.001). The least known determinants of infant gut microbiota were gestational BMI, prematurity, mode of delivery and NICU stay. Pregnant women failed to recognize the role of breastfeeding in the development of infant gut microbiota more frequently than non-pregnant women. The 97.5% of participants reported increased interest in the gut microbiota, with heightened interest associated with prior knowledge.

**Conclusions:**

Our study revealed a moderate level of knowledge about infant gut microbiota among respondents, emphasizing the positive impact of prior knowledge on understanding and interest. Targeted educational interventions are needed to address awareness gaps, especially concerning the influence of breastfeeding on infant gut microbiota. Healthcare providers have the potential to enhance women's knowledge and awareness of this topic.

**Supplementary Information:**

The online version contains supplementary material available at 10.1186/s12889-024-18497-8.

## Background

Humans are a complex ecosystem that thrives in symbiosis as long as the balance is maintained. The microorganisms living in and on the human body are collectively known as the human microbiota. Interestingly, the number of microorganisms exceeds that of human cells by an estimated factor of 10, with approximately 10 to 100 trillion microbes inhabiting the gastrointestinal tract alone [1].

Although it was initially presumed that the fetus grew in a sterile environment, an increasing body of evidence has challenged this foundational assumption. Indeed, among others, Collado and colleagues [2] documented an in utero microbial transfer by showing many similar taxonomic features between the placenta, amniotic fluid, and neonatal meconium in newborns delivered via caesarean section. Furthermore, Jimènez et al. [3] isolated commensal Gram^+^ bacteria from 9 out of 20 umbilical cord blood samples of newborns born by caesarean section after uneventful pregnancies. After this initial seeding, the development of the infant gut microbiota continues during the first years of life, in a 4-phase stepwise succession that leads to the establishment of an adult-like microbiota by 2 years of age [4]. Apart from genetic predisposition [5], many pregnancy-related, peri-partum and post-partum factors may influence the composition of the infant gut microbiota. It has been hypothesized that the administration of antibiotics during pregnancy, even in short courses like intrapartum antibiotic prophylaxis, may affect not only maternal but also neonatal microbiota [6, 7]. Likewise, a gestational high-fat diet [8] and increased maternal body mass index (BMI) [9, 10] appear to affect the development and composition of the infant gut microbiota. Mode of delivery is a known major influencing factor, as are prematurity and the often subsequent neonatal intensive care unit (NICU) hospitalization [11]. Among post-natal determinants of infant gut microbiota, human milk feeding plays a pivotal role [12], representing a continuous source of pre [13] and pro-biotics [14] for the nursing infant.

In recent years, the scientific community has manifested increasing interest in the human microbiota, and especially the gut microbiota, as emerging evidence suggests its profound influence on the host's long-term health. Recent studies have demonstrated that alterations in the composition and variability of the human microbiota can contribute to the development of various pathological conditions, ranging from immunological [15–18] to gastrointestinal [19, 20], metabolic [20–22], neurodevelopmental [23], neurological [24] and psychiatric [25] diseases.

Considering the crucial role of the gut microbiota in modulating long-term health and the multitude of factors that contribute to its development in the first 1000 days of life, it seems of utmost importance to promote health literacy regarding these topics in pregnant women and mothers of young children.

Health literacy refers to the ability to seek, understand, and use health information, empowering individuals to make informed decisions and take proactive steps to enhance their well-being. Low health literacy levels have been linked to adverse health outcomes, contributing to health inequalities and escalating healthcare costs [26]. However, despite the recognized importance of health literacy, healthcare providers are often unaware of patients’ health literacy levels.

Aim of the present study was to assess the knowledge of gut microbiota, its development and long-term impact among Italian pregnant women and mothers of children under 2 years old in order to identify potential knowledge gaps and risk factors for a low level of health literacy that may require additional support.

## Materials and methods

### Study design

This cross-sectional survey study aimed to assess the knowledge of Italian women regarding infant gut microbiota. The study employed an online self-administered questionnaire developed by a multidisciplinary team of neonatologists, healthcare assistants, and counselors with expertise in planned parenthood settings. Approval for the study was obtained from the Ethics Committee of the University of Milan (81/22, September 27th, 2022). The study was conducted in accordance with the Declaration of Helsinki. Informed consent to participate in the study was obtained from all study participants through a dedicated digital form, prominently displayed on the survey landing page. Participants were required to actively indicate their consent by checking a box before proceeding to the survey.

### Participant recruitment

The target audience comprised Italian pregnant women and mothers of children under 2 years old, to encompass a critical developmental period known as the first 1000 days of life, spanning from conception to the child's second birthday. This period was chosen for its recognized profound impact on health outcomes, including the crucial formation of the infant gut microbiota. Exclusion criteria were individuals outside this specified demographic, such as men, women with children older than 2 years old, and nulligravidas/nulliparas (G0P0), as our focus was on the early stages of motherhood. Other exclusion criteria were age < 18 years old, inadequate comprehension of the Italian language, and refusal to provide informed consent.

The survey was distributed through various social media channels (e.g., Facebook, WhatsApp, Instagram) between September 28th and November 15th, 2022. The social media platforms of the University of Milan were employed to reach a wide audience. Targeted posts, Instagram stories mentioning relevant hashtags and accounts, and direct messaging campaigns were employed. Specifically, Facebook groups and pages related to parenting and pregnancy, and WhatsApp groups of expectant or newly mothers were targeted. The use of a diverse range of recruitment channels aimed to enhance outreach and engage participants through different communication mediums, so as to include women with varying levels of interest or awareness, thus minimizing sampling and self-selection biases. The inclusion criteria were explicitly detailed within each social media post or direct messaging platform, ensuring transparency and clarity. These criteria were underscored across various posts to prevent any ambiguity or misunderstanding among potential participants regarding the study's eligibility requirements (i.e., Italian-speaking pregnant women and mothers of children under 2 years old).

Reminders were periodically posted to improve response rates.

### Instrument

The questionnaire (Suppl. Table 1) was created through an iterative process involving neonatologists, healthcare assistants, and planned parenthood counselors. A thorough literature review on gut microbiota, its determinants, and implications for long-term health informed the development process. The questionnaire was developed through multiple rounds of structured online and offline meetings to achieve consensus on the content and format of the questionnaire. The Google Forms platform (Google LLC, Mountain View, CA, USA) was employed for questionnaire creation, with a user-friendly format and clear instructions to limit response bias.

To ensure the clarity and comprehension of the questionnaire and address any potential ambiguity, a preliminary administration was conducted with 20 randomly selected women from the general population. Feedback from this sample was not included in the study, and the questionnaire remained unmodified as no issues arose during this phase.

The opening page of the survey featured an informed consent form, a brief overview of its contents, research objectives, and essential instructions for proper utilization.

Before engaging with the substantive questionnaire, participants were asked to provide information regarding their age, educational background, current occupation, pregnancy status, parity, previous knowledge of the gut microbiota ("Have you ever heard of the gut microbiota?"), and potential sources of information.

The questionnaire comprised 17 close-ended questions categorized into 3 domains:

1. Prenatal determinants of infant gut microbiota (5 questions);
2. Birth and neonatal determinants of infant gut microbiota (4 questions);
3. Health outcomes related to gut microbiota (8 questions).

These 3 domains were chosen to systematically cover a broad spectrum of topics, perceived by the Authors to be relevant for a comprehensive understanding of the infant gut microbiota. In designing the 17 questions, we considered both established associations and emerging research areas to provide a nuanced evaluation of maternal knowledge levels. Specifically, questions regarding fundamental knowledge assessed mothers' grasp of core concepts, while those addressing emerging topics evaluated the currency of their knowledge in light of recent research advancements. This approach aimed to provide a thorough assessment of maternal understanding of infant gut microbiota, encompassing both foundational principles and cutting-edge developments in the field. The questionnaire was anonymous and took approximately 15 min to complete. Participants could provide "yes," "no" or "I don’t know" responses. Each positive answer received a value of 1, while negative or "I don’t know" answers received a value of 0. A surrogate continuous variable, representing the sum of assigned values (ranging from 0 to 17), was created for each participant (total score).

After completing the questionnaire, participants were asked whether it had stimulated their interest in the gut microbiota.

All responses from the online survey were systematically recorded in an electronic spreadsheet and stored securely on password-protected servers accessible only to authorized personnel. To uphold participant anonymity, each participant was automatically assigned a consecutive alpha-numeric identifier and no personal information (e.g., names or contact details) was collected through the survey. Any identifying details mentioned in open-ended responses were promptly anonymized during data analysis.

### Statistical analysis

Continuous variables were reported as median and interquartile ranges, while categorical variables were expressed as frequencies and relative percentages. A Mann Whitney U test was used to compare continuous variables between groups, while a Chi Square test was used to identify potential differences in categorical variables between groups. A Pearson correlation test was used to identify the correlation coefficient between continuous variables. A Kruskal Wallis test was used to compare continuous variables between more than two groups.

To evaluate the strength of the association between sociodemographic characteristics and the total score, a linear regression analysis was performed using the total score as a dependent variable.

A sensitivity analysis was performed to assess the robustness of the study findings. Specifically, bootstrapping with 2000 samples and a 95%CI with corrected and accelerated bias was employed.Statistical analysis was performed with SPSS (Statistical Package for the Social Sciences) statistic software package (IBM SPSS Statistics for Windows, Version 25.0. Armonk, NY: IBM Corp.).

A *p*-value < 0.05 was considered statistically significant.

## Results

A total of 1076 women responded to the online questionnaire. The 17% (181) of them was pregnant at the time of the survey. Basic sociodemographic characteristics of the study population are summarized in Table 1.

**Table 1:** Basic characteristics of the study population divided by declared prior knowledge of gut microbiota

		Total population (*N* = 1076)	Have you ever heard of the gut microbiota		p
			**No (*N* = 196)**	**Yes (*N* = 880)**	
		**Median [IQR]; N (%)**	**Median [IQR]; N (%)**	**Median [IQR]; N (%)**	
Age, y		33 [31–36]	33 [30–35]	34 [31–37]	0.005
Level of education	**Primary school**	1 (0,1)	1 (0,5)	-	0.000
	**Secondary school**	32 (3,0)	13 (6,6)	19 (2,2)	
	**High school**	294 (27,3)	85 (43,4)	209 (23,7)	
	**University degree**	749 (69,6)	97 (49,5%)	652 (74,1%)	
Currently working	**No**	163 (15,1)	36 (18,4)	127 (14,4)	0.165
	**Yes**	913 (84,9)	160 (81,6)	753 (85,6)	
Parity	**Multiparous**	421 (39,1)	81 (41,3)	340 (38,6)	0.485
	**Primiparous**	655 (60,9)	115 (58,7)	540 (61,4)	
Currently pregnant	**No**	895 (83,2)	170 (86,7)	725 (82,4)	0.141
	**Yes**	181 (16,8)	26 (13,3)	155 (17,6)	

The studied population included women aged between 22 and 52 years old, with the 45% of the study sample falling into the 30–34 years age group. The majority of respondents had a university degree. Among the 913 working women, the 25.9% declared a healthcare-related profession.

The 81.7% of participants stated they had already heard of the gut microbiota. Women who had already heard of the gut microbiota were slightly older and had a university degree in a higher percentage of cases than women who had not. No differences between women who had already heard of the gut microbiota and those who had not were noted with regards to the other socio-demographic characteristics recorded.

**Fig. 1:**
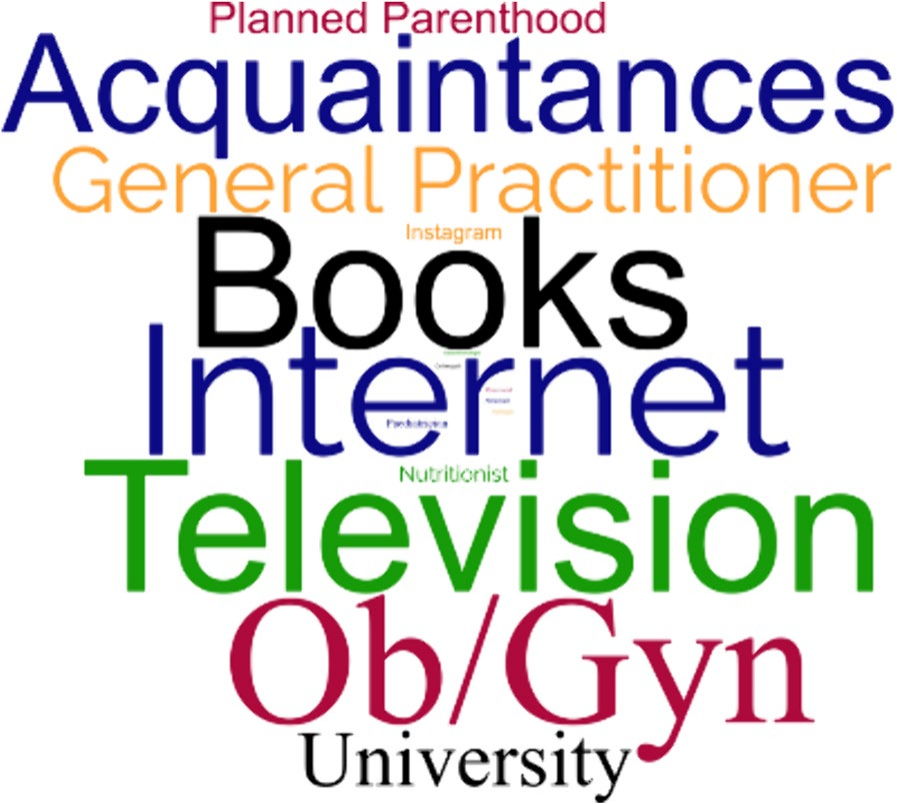
Graphical representation of word frequency (word cloud) based on the participants’ answers to the question “what was your primary source of information about the gut microbiota?”. The bigger and bolder the word appears, the more often it was mentioned by the study population

The relative frequencies of the declared sources of information on the gut microbiota are graphically represented by the word cloud in Fig. 1.

**Fig. 2:**
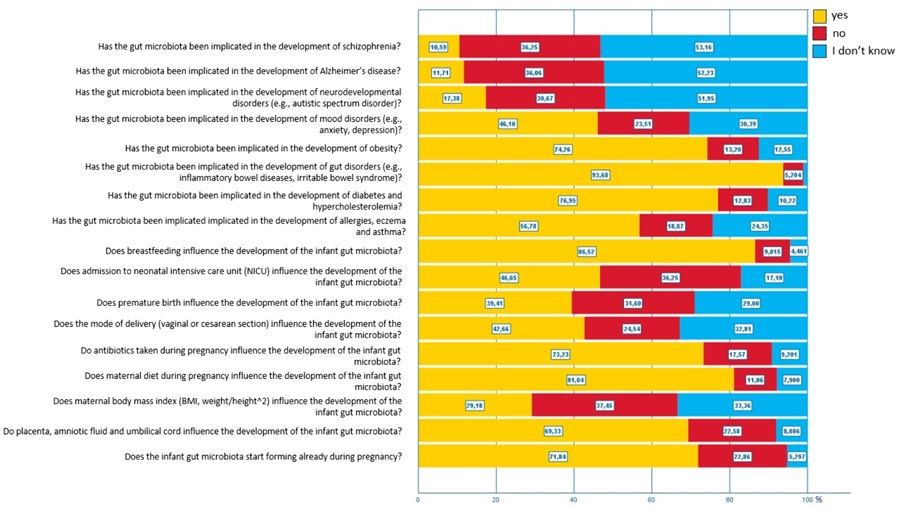
Relative frequencies of answers to the questionnaire in the total study population

Figure 2 shows the relative frequencies of the answers to the questions investigating women’s knowledge on the gut microbiota’s formation and long-term impact. The questionnaire showed acceptable internal consistency (Cronbach’s alpha = 0.74).

**Fig. 3:**
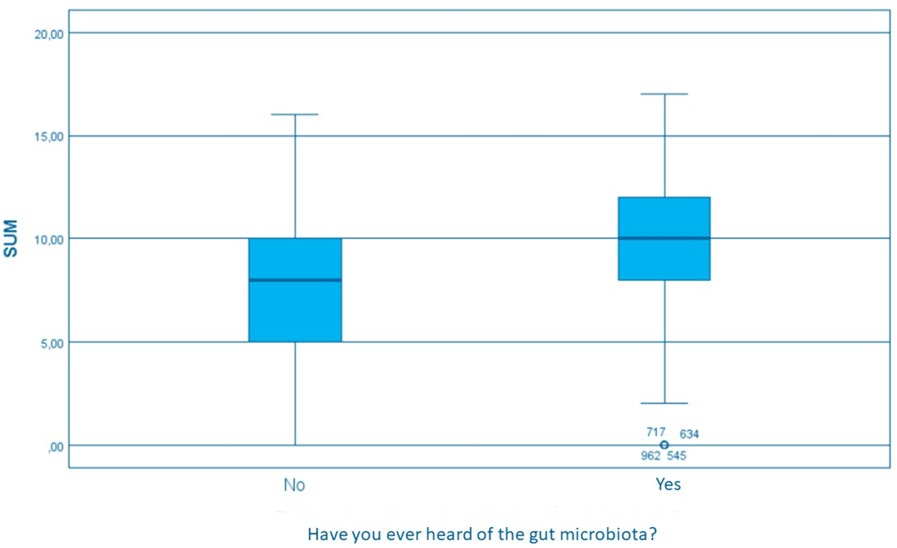
Box and whisker plot displaying distribution and skewness of the total score (SUM) among study participants divided by the answer to the question “Have you ever heard of the gut microbiota?”

Median total score was 9 [7-11]. Examining factors influencing knowledge scores, no difference in total score was found based on age, level of education, current occupation (working v. not working), pregnancy status (currently pregnant v. not currently pregnant), and parity. Conversely, women who reported a health-care-related occupation (median sum 11 v. 9, *p* = 0,0001) and women who had already heard of the gut microbiota (*p* < 0.001, Fig. 3) had higher total scores than their counterparts.

Women who had never heard of the gut microbiota failed to recognize the importance of perinatal factors on the formation of the infant gut microbiota more often than women who had previously heard of it. Likewise, women who had never heard of the gut microbiota were not aware of the impact of the gut microbiota on various health outcomes in a higher percentage of cases than women who had heard of it (Table 2).

**Table 2:** Relative frequencies of answers to the questionnaire divided by declared prior knowledge of gut microbiota

Question	Answer	Have you ever heard of the gut microbiota?		
		**No (*N* = 196)** **N (%)**	**Yes (*N* = 880)** **N (%)**	***p***
Does the infant gut microbiota start forming already during pregnancy?	No	7 (3,6%)	50 (5,7%)	< 0.001
	I don't know	92 (46,9%)	154 (17,5%)	
	Yes	97 (49,5%)	676 (76,8%)	
Do placenta, amniotic fluid and umbilical cord influence the development of the infant gut microbiota?	No	20 (10,2%)	67 (7,6%)	0.015
	I don't know	57 (29,1%)	186 (21,1%)	
	Yes	119 (60,7%)	627 (71,3%)	
Does maternal body mass index (BMI, weight/height^2) influence the development of the infant gut microbiota?	No	66 (33,7%)	293 (33,3%)	0.301
	I don't know	81 (41,3%)	322 (36,6%)	
	Yes	49 (25,0%)	265 (30,1%)	
Does maternal diet during pregnancy influence the development of the infant gut microbiota?	No	25 (12,8%)	60 (6,8%)	< 0.001
	I don't know	42 (21,4%)	77 (8,8%)	
	Yes	129 (65,8%)	743 (84,4%)	
Do antibiotics taken during pregnancy influence the development of the infant gut microbiota?	No	25 (12,8%)	74 (8,4%)	0.012
	I don't know	44 (22,4%)	145 (16,5%)	
	Yes	127 (64,8%)	661 (75,1%)	
Does the mode of delivery (vaginal or cesarean section) influence the development of the infant gut microbiota?	No	104 (53,1%)	249 (28,3%)	< 0.001
	I don't know	55 (28,1%)	209 (23,8%)	
	Yes	37 (18,8%)	422 (47,9%)	
Does premature birth influence the development of the infant gut microbiota?	No	75 (38,3%)	237 (26,9%)	< 0.001
	I don't know	70 (35,7%)	270 (30,7%)	
	Yes	51 (26,0%)	373 (42,4%)	
Does admission to neonatal intensive care unit (NICU) influence the development of the infant gut microbiota?	No	46 (23,5%)	138 (15,7%)	0.003
	I don't know	78 (39,8%)	312 (35,4%)	
	Yes	72 (36,7%)	430 (48,9%)	
Does breastfeeding influence the development of the infant gut microbiota?	No	18 (9,2%)	30 (3,4%)	< 0.001
	I don't know	40 (20,4%)	57 (6,5%)	
	Yes	138 (70,4%)	793 (90,1%)	
Has the gut microbiota been implicated in the development of allergies, eczema and asthma?	No	58 (29,6%)	204 (23,2%)	< 0.001
	I don't know	51 (26%)	152 (17,3%)	
	Yes	87 (44,4%)	524 (59,5%)	
Has the gut microbiota been implicated in the development of diabetes and hypercholesterolemia?	No	26 (13,3%)	84 (9,5%)	0.001
	I don't know	39 (19,9%)	99 (11,3%)	
	Yes	131 (66,8%)	697 (79,2%)	
Has the gut microbiota been implicated in the development of intestinal disorders (e.g., inflammatory bowel diseases, irritable bowel syndrome)?	No	2 (1,0%)	10 (1,1%)	< 0.001
	I don't know	28 (14,3%)	28 (3,2%)	
	Yes	166 (84,7%)	842 (95,7%)	
Has the gut microbiota been implicated in the development of obesity?	No	24 (12,2%)	111 (12,6%)	0.001
	I don't know	42 (21,4%)	100 (11,4%)	
	Yes	130 (66,3%)	669 (76,0%)	
Has the gut microbiota been implicated in the development of mood disorders (e.g., anxiety, depression)?	No	71 (36,2%)	256 (29,1%)	< 0.001
	I don't know	60 (30,6%)	193 (21,9%)	
	Yes	65 (33,2%)	431 (49,0%)	
Has the gut microbiota been implicated in the development of neurodevelopmental disorders (e.g., autistic spectrum disorder)?	No	105 (53,6%)	454 (51,6%)	0.031
	I don't know	69 (35,2%)	261 (29,6%)	
	Yes	22 (11,2%)	165 (18,8%)	
Has the gut microbiota been implicated in the development of Alzheimer’s disease?	No	103 (52,6%)	459 (52,2%)	0.016
	I don't know	81 (41,3%)	307 (34,9%)	
	Yes	12 (6,1%)	114 (12,9%)	
Has the gut microbiota been implicated in the development of schizophrenia?	No	102 (52,0%)	470 (53,4%)	0.212
	I don't know	79 (40,3%)	311 (35,3%)	
	Yes	15 (7,7%)	99 (11,3%)	

Furthermore, women who had never heard of the gut microbiota answered “I don’t know” in a higher percentage of cases than women who had already heard of it (*p* = 0.001).

Regarding current pregnancy status, the study revealed that pregnant women showed lower awareness compared to non-pregnant women regarding the potential impact of gestational age at birth on infant gut microbiota (30.0% vs. 41.2%, *p* = 0.010). Moreover, non-pregnant women displayed greater awareness than pregnant women regarding the role of breastfeeding in microbiota formation (87.8% vs. 80.1%, *p* = 0.014). No significant differences between this two groups were observed with regards to the other questions.

The 97.5% of participants declared that the questionnaire stimulated their interest in the gut microbiota. Those who reported increased interest were more likely to have prior knowledge of the gut microbiota (82.2% vs. 66.7%, *p* = 0.039).

To explore factors independently associated with microbiota-related health literacy, a multivariable regression analysis was conducted (Table 3). Noteworthy predictors of higher total score (used as a proxy of maternal general knowledge of the infant gut microbiota) included having a university degree (B = 0.656, *p* = 0.002) and prior knowledge of the gut microbiota (B = 2.246, *p* < 0.001). Conversely, older age was associated with lower total scores (B = -0.092, *p* < 0.001). The sensitivity analysis showed consistent significance levels.

**Table 3:** Independent predictors of total score identified at multivariate logistic regression analysis, including unstandardized coefficients (B), standard errors (SE), standardized coefficients (Beta), t-values, *p*-values, and 95% confidence intervals (CI) for each predictor variable

Predictor Variable	B	SE	Beta	t	*p*-value	95%CI for B
(Constant)	8.248	0.925	-	8.916	0.000	[6.433, 10.063]
Pregnancy status	-0.383	0.250	-0.045	-1.528	0.127	[-0.874, 0.109]
Healthcare-related occupation	0.008	0.039	0.006	0.209	0.835	[-0.069, 0.085]
University degree	0.656	0.214	0.094	3.067	0.002	[0.236, 1.075]
Prior knowledge	2.246	0.247	0.271	9.087	0.000	[1.761, 2.730]
Age	-0.092	0.023	-0.119	-3.996	0.000	[-0.137, -0.047]

## Discussion

The development of a questionnaire about the infant gut microbiota stemmed from the acknowledgement of its pivotal role in long-term health and the belief that understanding the factors influencing its composition and the potential consequences of dysbiosis is crucial for maternal and child well-being. Based on such premises, the questionnaire sought to evaluate maternal gut microbiota-related health literacy and identify knowledge gaps, enabling targeted educational interventions and public health campaigns to enhance maternal awareness and contribute to the health outcomes of both mothers and their children.

The demographic characteristics of our study population align with data observed in the general population. Indeed, the Italian National Institute of Statistics (ISTAT) and the Ministry of Health (Certificato di Assistenza al Parto—CeDAP) reported a mean maternal age at birth of around 33 years in 2022 [27, 28]. However, the prevalence of university degrees and employment in our study population exceeds that of the general Italian female population. According to ISTAT [29], 35.5% of women aged 25 to 34 hold a university degree, while the employment rate among Italian women is approximately 60%. Notably, university graduates exhibit an employment rate 18.4 points higher than high school graduates, who, in turn, have a 25.8-point advantage over women with a secondary school education [29]. After becoming mothers, 16% of university and high school graduates stop working, compared to 21% of mothers with a secondary school education [30]. This disparity likely contributed to the elevated employment rate observed in our population.

Our study reported a moderate level of knowledge on infant gut microbiota among respondents. A recent cross-sectional survey study on Turkish pregnant women [31] evaluated participants' understanding of the intestinal microbiota, pre- and probiotics through 20 Likert-scale questions. Five of those questions align with our study, investigating participants' knowledge on the timing of infant gut microbiota formation, the role of breastfeeding in its development, and the impact of intestinal microbiota on obesity, diabetes, Alzheimer's disease, and depression. The study revealed acceptable general knowledge (especially in terms of timing of onset of gut microbiota formation in the womb and pivotal role of diet in its development) but low awareness of disease-microbiota relationships. Regrettably, given the content disparities between the questionnaire used and ours, direct result comparisons are not feasible.

In our study, prior knowledge emerged as an independent predictor of higher total scores. Mothers already familiar with microbiota concepts exhibited a more comprehensive understanding of the factors influencing its development and the potential consequences of dysbiosis. Conversely, those lacking familiarity were found to be more frequently unaware of significant factors, such as the impact of maternal diet during pregnancy and breastfeeding. This highlights the crucial role of pre-existing knowledge in shaping awareness and at the same time underscores the need for targeted interventions for those lacking foundational information, considering that, although prior knowledge is a strong determinant of learning success, it is not the only one [32]. The link between prior knowledge and greater understanding is unsurprising and in line with common expectations. A previous study aimed at evaluating microbiota knowledge among university students in Jordan found that those who had taken a microbiology course exhibited significantly higher microbiota knowledge scores and greater awareness of the impact of antibiotics on microbiota compared to students with self-reported basic or poor knowledge of the subject [33].

Other independent predictors of greater knowledge found in our study were level of education (i.e., university degree) and younger age, in line with what has been previously reported in a survey study conducted in the United Arab Emirates [34].

The diversity of primary information sources reported by respondents, including the internet, books, television, Ob/Gyn, and acquaintances among the most frequently referred to, underscores the wide range of channels through which mothers nowadays seek information about infant gut microbiota, reflecting the influence of both traditional and digital platforms. In the last decades the accessibility of online information has seen a rapid increase, even among individuals with limited literacy skills. As a matter of fact, in this digital era, a new concept has been introduced: *eHealth literacy*, that is an individual's ability to seek, find, understand, and appraise health information from electronic sources and apply the knowledge gained to address or solve a health problem [35]. Unfortunately, the internet may be a double-edged sword and the recent surge in often well-intentioned, yet inaccurate health resources online may pose a threat to public health. Indeed, particularly through social media, patients may encounter information that is incomplete or incorrect. When individuals are exposed to misinformation that aligns with their worldview, is widespread and comes from a source perceived to be trustworthy, they may resist accepting accurate information, even in the face of striking evidence. Accumulating such misinformation can lead to flawed judgments, resulting in harmful health choices. Consequently, misinformation has emerged as a distinct form of health literacy failure, posing risks as significant as, if not greater than, the absence of information [36]. The diverse set of sources reported in the present study highlights the need for comprehensive health communication strategies that consider the multitude of channels through which women access information on infant gut microbiota, to interrupt the vicious cycle of misinformation and misguided decision-making. These targeted interventions should focus on providing accurate, evidence-based information on the infant gut microbiota and related health topics through validated, perinatal-focused health communication materials or through strictly fact-checked Institutional social-media accounts. Strategies for enhancing gut microbiota-related health literacy among mothers may also include the development of educational campaigns tailored to specific demographic groups (e.g., pregnant women), and the promotion of partnerships between healthcare providers and community organizations to disseminate accurate information through community-based interventions [37] aimed at empowering mothers to make informed decisions about their health and the health of their children.

The present study identified specific questions that were frequently answered incorrectly, revealing areas of misconception and information gaps. The questions most frequently answered incorrectly by women participating in the survey were those related to the impact of maternal gestational BMI, prematurity, mode of delivery and NICU stay on the infant gut microbiota, and those concerning the potential influence of gut microbiota on neurological, neurodevelopmental, or psychiatric disorders. While it is understandable that the latter may be less known (also considering the uncertainty that still surrounds these aspects), it is concerning that the former are not widely recognized. In particular, we believe that the impact of cesarean section, known and ascertained risk factor for altered infant gut microbiota formation [11], should be common knowledge among women and should be part of the information routinely provided during Ob/Gyn consultations. Likewise, women should be correctly informed about the additional perils an excessive increase of weight during pregnancy carries for the infant gut microbiota [9].

Moreover, in our study population, pregnant women exhibited less awareness than non-pregnant women on two important influencing factors of the infant gut microbiota: prematurity and breastfeeding. The latter seems particularly worrisome, since, compared to the former, it is a modifiable factor. After birth, breastfeeding offers numerous benefits for the newborn, contributing to optimal growth and cognitive development. Additionally, Stewart et al. [12] identified breastfeeding as the most important variable explaining infant gut microbiota composition out of 22 variables tested in a cohort of European and American infants. Promoting maternal knowledge of this important, albeit often overlooked, role of breast milk may ultimately help boost breastfeeding rates.

Among respondents, the declared level of interest for the gut microbiota was generally high, and this study showed that the more mothers know about it, the more they want to know. The eagerness of the majority of women to expand their understanding of gut microbiota underscores the need for healthcare providers to proactively offer information and counseling on this topic. However, it is not always easy or even feasible to balance the complexity and volume of health-promotion information within the time constraints of each Ob/Gyn or pediatric office visit. Innovative strategies are therefore needed to deliver essential information effectively, such as jargon-free take-home print materials [37].

The present study has several notable strengths that make it a valuable contribution to the understanding of mothers’ knowledge about the infant gut microbiota. The questionnaire used for the purposes of the present study was developed by a multidisciplinary team of neonatologists, healthcare assistants, and planned parenthood counselors, reflecting a comprehensive approach to questionnaire design. This collaboration ensured a well-rounded and nuanced exploration of the topic. Moreover, the study achieved a robust sample size, providing a diverse and sizable dataset. This enhances the generalizability of the findings to a broader population of women in similar demographics.

However, there are some limitations that need to be acknowledged for a correct interpretation of study results. First of all, the questionnaire we used has not been validated. Consequently, it may lack proven accuracy and reliability in measuring respondents’ knowledge about the infant gut microbiota. Moreover, although our questionnaire was preliminary tested to assess comprehension and eventual ambiguities, a residual risk of misinterpretation or misunderstanding of questions cannot be excluded. It should also be acknowledged that the present questionnaire reflects the inherent challenges of formulating questions on a dynamic research topic. Guided by available evidence and multidisciplinary expertise, the questions were designed to capture participants' awareness and provide a comprehensive understanding of their knowledge, even in the absence of universally agreed-upon right or wrong answers. However, the rapidly evolving nature of research on gut microbiota may affect the accuracy of our study's findings over time, since our questionnaire may quickly become outdated. Nevertheless, we believe that our study can still provide a valid snapshot of the current state of knowledge among Italian pregnant women and mothers, according to the current understanding of gut microbiota.

As the survey was distributed through social media channels, there may have been a bias towards individuals who use social media more frequently, potentially resulting in a sampling bias. Moreover, participants who chose to take the questionnaire may have had a higher interest or awareness of the topic compared to those who opted not to participate. Likewise, individuals who chose not to participate might differ systematically from those who participated, thus potentially introducing an additional non-responder bias. However, the survey's distribution methods prevent accurate quantification of the total recipients and collection of their sociodemographic information, making an exact response rate calculation and characterization of non-responders not feasible. Moreover, as is often the case in survey studies, we cannot exclude the occurrence of self-report and social desirability biases leading to misrepresentation of participants' knowledge levels and thus impacting our findings.

Furthermore, it is important to acknowledge the overrepresentation of university graduates within our study population compared to the general Italian population. This skew towards higher education levels may limit the generalizability of our findings to the broader Italian population, as our findings may not fully reflect the knowledge of gut microbiota of women with different educational backgrounds. Moreover, our study is a preliminary evaluation of current knowledge of gut microbiota among Italian mothers, albeit with the aforementioned limitations, and thus its results cannot be generalized to other cultural contexts or populations. Future research should aim to diversify participants demographics, also considering the geographical origin and socio-economic background of the participants. These factors, which were not accounted for in the current study, could potentially influence study outcomes.

Finally, future longitudinal studies should explore the effectiveness of different educational strategies in enhancing maternal knowledge of gut microbiota, and assess how this, in turn, may influence health-related behaviors, such as breastfeeding duration, dietary choices, and antibiotic use, and ultimately impact children's long term health outcomes.

## Conclusions

This study sheds light on the current state of Italian mothers’ knowledge on infant gut microbiota and advocates for dedicated discussions led by healthcare professionals to ensure accurate information and mitigate the risks of misinformation from less-qualified sources. Specific misconceptions regarding the impact of modifiable factors on infant gut microbiota were identified, signaling areas for targeted educational initiatives. Additionally, the study reaffirms the importance of emphasizing breastfeeding as a key component in promoting infant health.

### Supplementary Information

**Additional file 1: Suppl. Table 1.** Online questionnaire on the infant gut microbiota.

## Data Availability

The dataset used and analysed during the current study is available from the corresponding author on reasonable request.

## Abbreviations

BMI: Body Mass Index
NICU: Neonatal Intensive Care Unit
Ob/Gyn: Obstetrician/Gynecologist
