# Supplementary material for: From womb to world: mapping gut microbiota-related health literacy among Italian mothers, a cross-sectional study
Source: BMC Public Health. 2024 Apr 12;24:1012. doi: 10.1186/s12889-024-18497-8 (PMC11010343; doi:10.1186/s12889-024-18497-8)
Supplement: Supplementary file 1 — Additional file 1: Suppl. Table 1. Online questionnaire on the infant gut microbiota. [file 12889_2024_18497_MOESM1_ESM.docx]

| Does the infant gut microbiota start forming already during pregnancy? | YES |
| --- | --- |
|  | **NO** |
|  | **I DON’T KNOW** |
| Do placenta, amniotic fluid and umbilical cord influence the development of the infant gut microbiota? | **YES** |
|  | **NO** |
|  | **I DON’T KNOW** |
| Does maternal body mass index (BMI, weight/height^2) influence the development of the infant gut microbiota? | **YES** |
|  | **NO** |
|  | **I DON’T KNOW** |
| Does maternal diet during pregnancy influence the development of the infant gut microbiota? | **YES** |
|  | **NO** |
|  | **I DON’T KNOW** |
| Do antibiotics taken during pregnancy influence the development of the infant gut microbiota? | **YES** |
|  | **NO** |
|  | **I DON’T KNOW** |
| Does the mode of delivery (vaginal or cesarean section) influence the development of the infant gut microbiota? | **YES** |
|  | **NO** |
|  | **I DON’T KNOW** |
| Does premature birth influence the development of the infant gut microbiota? | **YES** |
|  | **NO** |
|  | **I DON’T KNOW** |
| Does admission to neonatal intensive care unit (NICU) influence the development of the infant gut microbiota? | **YES** |
|  | **NO** |
|  | **I DON’T KNOW** |
| Does breastfeeding influence the development of the infant gut microbiota? | **YES** |
|  | **NO** |
|  | **I DON’T KNOW** |
| Has the gut microbiota been implicated in the development of allergies, eczema and asthma? | **YES** |
|  | **NO** |
|  | **I DON’T KNOW** |
| Has the gut microbiota been implicated in the development of diabetes and hypercholesterolemia? | **YES** |
|  | **NO** |
|  | **I DON’T KNOW** |
| Has the gut microbiota been implicated in the development of intestinal disorders (e.g., inflammatory bowel diseases, irritable bowel syndrome)? | **YES** |
|  | **NO** |
|  | **I DON’T KNOW** |
| Has the gut microbiota been implicated in the development of obesity? | **YES** |
|  | **NO** |
|  | **I DON’T KNOW** |
| Has the gut microbiota been implicated in the development of mood disorders (e.g., anxiety, depression)? | **YES** |
|  | **NO** |
|  | **I DON’T KNOW** |
| Has the gut microbiota been implicated in the development of neurodevelopmental disorders (e.g., autistic spectrum disorder)? | **YES** |
|  | **NO** |
|  | **I DON’T KNOW** |
| Has the gut microbiota been implicated in the development of Alzheimer’s disease? | **YES** |
|  | **NO** |
|  | **I DON’T KNOW** |
| Has the gut microbiota been implicated in the development of schizophrenia? | **YES** |
|  | **NO** |
|  | **I DON’T KNOW** |
